# Laparoscopic bilateral cervicosacropexy: introduction to a new tunneling technique

**DOI:** 10.1007/s00192-019-03911-2

**Published:** 2019-03-08

**Authors:** Sebastian Ludwig, Bernd Morgenstern, Peter Mallmann, Wolfram Jäger

**Affiliations:** Faculty of Medicine and University Hospital of Cologne, Department of Gynecology and Obstetrics, University of Cologne, Kerpenerstrasse 34, 50931 Köln, Germany

**Keywords:** laCESA, Mixed urinary incontinence, Pelvic organ prolapse, Polyvinylidene fluoride, Uterosacral ligament replacement, Urgency urinary incontinence, Video tutorial

## Abstract

**Introduction and hypothesis:**

To elevate and suspend the apical end of the vagina, the uterosacral ligaments (USL) were replaced by polyvinylidene fluoride (PVDF) structures. These PVDF structures were placed in the peritoneal folds of the USL at the pelvic wall to mimic the lateral and backward tension and to avoid rectal obstruction. A special tunneling device was used, which allowed the semi-circular placement of the structure without destroying the peritoneum.

**Methods:**

A 59-year-old woman with mixed urinary incontinence and apical prolapse (pelvic organ prolapse quantification system, POP-Q, stage 2) of the uterus underwent laparoscopic bilateral USL replacement. USLs were replaced by PVDF structures by performing the cervicosacropexy (CESA) technique using a semi-circular tunneling device.

**Results:**

Apical support was restored (POP-Q stage 0), and the patient was continent thereafter. The tunneling device was pulled through the peritoneal folds of the USLs toward the cervix. The new USL structures were brought to their physiological position. The new technique did not lead to any complications and did not cause any side effects during 1-year follow-up.

**Conclusions:**

Restoration of apical prolapse and urinary continence was achieved by bilateral USL replacement using a semi-circular tunneling device that was inserted through the lateral abdominal trocar incision.

**Electronic supplementary material:**

The online version of this article (10.1007/s00192-019-03911-2) contains supplementary material. This video is also available to watch on http://link.springer.com/. Please search for this article by the article title or DOI number, and on the article page click on ‘Supplementary Material’

## Introduction

In 2012, Jäger et al. introduced a new method to replace the uterosacral ligaments (USL) bilaterally, called cervicosacropexy (CESA) and vaginosacropexy, to restore the physiological fixation of the apical vagina [1]. Thus, the anterior vaginal wall was elevated, supporting the bladder base and bladder neck. Notably, this surgical procedure led to continence in a considerable number of women suffering from mixed and urgency urinary incontinence (MUI and UUI respectively) [2–4].

To investigate this “side effect,” the surgical procedure was standardized by utilizing polyvinylidene fluoride (PVDF) structures of identical length for USL replacement at identical anatomical fixation sides. This was possible because the bony dimensions of the small pelvis are nearly identical among women of different ethnic groups [5].

The peritoneal folds of the USL run semi-circularly on both sides of the bony pelvis. The backward tension also leads to lateral tension, which protects the bowel from obstruction. To place the new USL structures in these folds, an adjusted surgical device is required. A semi-circular curved tunneling device offered the best qualities.

This video is aimed at demonstrating the use of this semi-circular device for bilateral USL replacement.

## Materials and methods

A 59-year-old woman with MUI and apical prolapse was referred to our tertiary urogynecological unit. She lost urine most of the time before she could get to the toilet and leaked urine when she was physically active. These symptoms occurred every day (International Consultation on Incontinence Questionnaire, ICIQ-FLUTS, score 14). Preoperative urodynamics revealed detrusor overactivity. Conservative treatment options failed (pelvic floor exercise and anticholinergics), and she had not undergone previous urogynecological surgery.

Vaginal examination was performed in the dorsal lithotomy and standing positions. The respective anatomical points were measured according to the POP-Q system [6].

Approval for the development of a laparoscopic approach to CESA was obtained from our clinical directors through a conference and the Ethics Committee of the Medical Faculty of the University of Cologne, Germany (Approval No. 11–016). Written informed consent was obtained from the patient before laparoscopic cervicosacropexy (laCESA).

For laCESA, the patient was positioned in the dorsal lithotomy position. After induction of general anesthesia, a gynecological examination was performed, and two clamps were placed at the cervix at the 3 and 9 o’clock positions to manipulate the uterus. The patient was maintained in a head-down position of 25°. CO_2_ pneumoperitoneum was conducted according to institutional standards, and four trocars were placed. Once intraperitoneal pressure reached 12 mmHg, a 10-mm optical trocar was inserted through the umbilicus. Another 10-mm trocar was placed 1 cm above the symphysis pubis in the midline. Two 5-mm lower quadrant trocars were placed lateral to the rectus abdominis muscles and 3 cm medial to the anterior–superior iliac spine.

The origin of both USL (at the posterior cervix) was identified by elevating the uterus. The attachment of both USL (at the sacral vertebra in front of S1) was identified by the lateralization of the rectosigmoid colon. Subsequent steps were as follows:

1. Subtotal hysterectomy was performed above the origin of the USL at the posterior cervix and at the peritoneal fold of the bladder peritoneum at the anterior cervix.
2. The lateral peritoneum over the first sacral vertebra was incised for 1.5 cm on either side of the rectosigmoid colon; consequently, the ureters and iliac vessels were located laterally, and the hypogastric nerve was spared.
3. For USL replacement, a PVDF structure measuring 8.8 cm in length and 4 mm in width was used (Dynamesh CESA; FEG, Aachen, Germany). As a tunneling device, a semi-circular curved hook with a handle measuring 22 cm in length and a radius of 4.5 cm was inserted via the right lateral trocar incision (by removing the right trocar).
4. The blunt tip of the semi-circular tunneler was inserted into the right sacral peritoneal window and advanced under the peritoneum along the length of the right USL toward the cervix. The lateral end of the PVDF structure was threaded through the hole of the tunneling device’s tip, and the tunneler was then pulled back. On the left side, the rectosigmoid colon was pulled to the right side, the left ureter and iliac vessels were identified, and the tunneler’s blunt tip was inserted into the left sacral peritoneal window and advanced under the peritoneum along the length of the left USL.
5. The central fixation part of the PVDF structure was sutured to all quadrants in the cut surface on the cervix by using four interrupted non-absorbable sutures (Ethibond; Ethicon, Somerville, NJ, USA).
6. Each arm of the PVDF structure was attached (at defined locations at the PVDF structures to establish defined “tension” of the new USLs) with three titanium helices to the right and left lateral prevertebral fascia of S1 by using a fixation device (ProTack; Covidien, Mansfield, MA, USA).
7. Finally, the peritoneum above the cervix was closed by using a running suture (V-Loc 180 absorbable; Covidien, Mansfield, MA, USA).

## Results

In accordance with the CESA laparotomy, we used a laparoscopic approach to replace the USL in the current patient. The integrity of the peritoneal USL folds remained intact after the PVDF structures were placed. The overall operation time was 78 min. No major complications occurred.

After surgery, no prolapse was detected (POP-Q stage 0) and the patient was continent again (no involuntary loss of urine, ICIQ-FLUTS score 0). At 1-year follow-up, the patient still had POP-Q stage 0 and did not report any urinary incontinence symptoms (preoperative—dorsal lithotomy/standing: Aa −3/−2; Ba −2/−1; C −3/0; total vaginal length (TVL) 9/9; Ap −3/−3; Bp −3/−3; postoperative—Aa −3; Ba −3; C −8; TVL 9; Ap −3; Bp −3). Detrusor overactivity was not detectable. No complications of the implanted PVDF structure or other major side effects were observed.

## Discussion

The apical end of the vagina has attracted interest in terms of surgical interventions. The upper half of the anterior vaginal wall supports the bladder and the vesico-urethral junction, especially in the upright body position. It has been shown that prolapse operations can lead to continence in patients with MUI and UUI [7, 8]. This can be explained by the elevation of the anterior vaginal wall, which causes activation of stretch receptors in the bladder [9].

The CESA operation is a standardized procedure to re-establish the physiological fixation of the cervix, thereby elevating the anterior vaginal wall. A nearly identical tensioning of the vaginal apical end is achieved in all patients because of the identical length (8.8 cm) of the implanted USL structures.

Surgical instruments for laparoscopy are usually straight because they are inserted via the rigid trocar. These straight instruments turned out to be inconvenient for placing the USL structure into the semi-circular peritoneal USL fold. Using a curved instrument for a laparoscopic procedure might seem strange; however, we decided to use a semi-circular tunneling device that could easily be inserted via the lateral trocar incision.

After inserting the tunneler’s tip into the peritoneal folds of the USL, it can be seen gliding below the peritoneum to the lateral cervix—on both sides of the small pelvis. Thereby, major injuries to the surrounding vessels, ureters, and nerves can be prevented.

## Conclusion

The advantage of this laparoscopic procedure for bilateral USL replacement lies in its clearly defined surgical technique and the minimal amount of synthetic material used. The curved tunneling device simplified the placement of the PVDF structures into the semi-circular peritoneal fold of the USLs.

## Electronic supplementary material

ESM 1 (MP4 97,189 kb)
